# Quantitative sleep electroencephalogram and cognitive performance in Parkinson’s disease with and without rapid eye movement sleep behavior disorder

**DOI:** 10.3389/fneur.2023.1223974

**Published:** 2023-09-07

**Authors:** Adeel A. Memon, Corina Catiul, Zachary Irwin, Jennifer Pilkington, Raima A. Memon, Allen Joop, Kimberly H. Wood, Gary Cutter, Svjetlana Miocinovic, Amy W. Amara

**Affiliations:** ^1^Department of Neurology, University of Alabama at Birmingham, Birmingham, AL, United States; ^2^Department of Neurology, West Virginia University, Morgantown, WV, United States; ^3^Department of Neurosurgery, University of Alabama at Birmingham, Birmingham, AL, United States; ^4^Department of Pathology, University of Alabama at Birmingham, Birmingham, AL, United States; ^5^Department of Psychology, Samford University, Birmingham, AL, United States; ^6^Department of Biostatistics, University of Alabama at Birmingham, Birmingham, AL, United States; ^7^Department of Neurology, Emory University, Atlanta, GA, United States; ^8^Department of Neurology, University of Colorado, Anschutz Medical Campus, Aurora, CO, United States

**Keywords:** Parkinson’s disease, quantitative sleep neurophysiology, non-rapid eye movement sleep, scalp slow wave, sleep spindles, phase-amplitude coupling, REM sleep behavior disorder, cognition

## Abstract

**Introduction:**

Parkinson’s disease (PD) patients with REM sleep behavior disorder (RBD) are at greater risk for cognitive decline and RBD has been associated with alterations in sleep-related EEG oscillations. This study evaluates differences in sleep quantitative EEG (qEEG) and cognition in PD participants with (PD-RBD) and without RBD (PD-no-RBD).

**Methods:**

In this cross-sectional study, polysomnography (PSG)-derived qEEG and a comprehensive level II neuropsychological assessment were compared between PD-RBD (*n* = 21) and PD-no-RBD (*n* = 31). Following artifact rejection, qEEG analysis was performed in the frontal and central leads. Measures included Scalp-slow wave (SW) density, spindle density, morphological properties of SW and sleep spindles, SW-spindle phase-amplitude coupling, and spectral power analysis in NREM and REM. The neurocognitive battery had at least two tests per domain, covering five cognitive domains as recommended by the Movement Disorders Society Task Force for PD-MCI diagnosis. Differences in qEEG features and cognitive performance were compared between the two groups. Stepwise linear regression was performed to evaluate predictors of cognitive performance. Multiple comparisons were corrected using the Benjamini-Hochberg method.

**Results:**

Spindle density and SW-spindle co-occurrence percent were lower in participants with PD-RBD compared to PD-no-RBD. The PD-RBD group also demonstrated higher theta spectral power during REM. Sleep spindles and years of education, but not RBD, were predictors of cognitive performance.

**Conclusion:**

PD participants with RBD have alterations in sleep-related qEEG compared to PD participants without RBD. Although PD-RBD participants had worse cognitive performance compared to PD-no-RBD, regression models suggest that lower sleep spindle density, rather than presence of RBD, predicts worse comprehensive cognitive score. Future studies should include longitudinal evaluation to determine whether sleep-related qEEG alterations are associated with more rapid cognitive decline in PD-RBD.

## Introduction

The sleep cycle serves a variety of vital functions throughout an individual’s lifespan and is made up of distinct stages: rapid eye movement (REM) and non-rapid eye movement (NREM) (1). Every stage of sleep is governed by a distinct pattern of electrophysiological rhythms. These rhythms are severely compromised in the presence of neurodegenerative diseases such as idiopathic REM sleep behavior disorder (iRBD) (2, 3) and Parkinson’s disease (PD) (4). RBD is a parasomnia characterized by a loss of muscle atonia and complex motor behaviors during REM sleep (5) and is common in PD, with a prevalence of 16–47% (6). Furthermore, RBD is recognized as a prodromal stage of synucleinopathies. Mounting evidence indicates that PD patients with RBD (PD-RBD) suffer from greater cognitive impairment compared to PD patients without RBD (PD-no-RBD) (7, 8). However, the mechanism underlying this phenomenon remains unclear.

Quantitative sleep EEG (qEEG) measures are neurophysiological markers that can inform the pathophysiological mechanisms underpinning cognitive performance in older adults (9) and those with neurodegenerative disorders (10). Sleep-related slow wave activity (SWA, 0.5–4 Hz) is detectable via scalp EEG during deep sleep and occurs predominantly during NREM stage 3 (N3) (11, 12). There are two components to this EEG pattern, namely, the Scalp-Slow wave (SW) (<1 Hz) and delta power (1.0–4 Hz) (13). Among other sleep-related oscillations that contribute to cognition are sleep spindles (9), which are characteristic of the EEG in NREM stage 2 (N2), the phase-amplitude relationship between SW and spindles (11), and the spectral power of REM and NREM sleep (14–16). Prior work suggests that patients with iRBD have a less steep SW slope (2), reduced spindle density (3), and impaired SW-spindle coupling (2), as well as greater power in the delta and theta frequency bands during REM sleep (17). Furthermore, slow and fast sleep spindles play crucial roles in sleep architecture and cognitive processes. Slow spindles (typically defined as <12 Hz) are associated with memory consolidation, learning, and information transfer between brain regions (14). On the other hand, fast spindles (approximately >12 Hz) are believed to contribute to sleep stability and cortical synchronization, promoting efficient sleep maintenance and quality (14). In addition, sleep spindles in N2 sleep have lower frequency and shorter duration compared to N3 sleep, where they exhibit higher frequency and longer duration (18). However, to the best of our knowledge, no study to date has assessed the differences in quantitative sleep EEG markers between PD patients with RBD (PD-RBD) and those without RBD (PD-no-RBD).

Toward this aim, a laboratory-based polysomnography-derived scalp EEG and a comprehensive level II neurocognitive assessment were used to evaluate NREM and REM qEEG and cognition in PD patients with and without RBD. Specifically, we investigated the hypothesis that PD patients with RBD would have lower SW and spindle densities, a reduced SW-spindle co-occurrence percent, higher power in the delta and theta frequency bands during REM, and poorer performance on cognitive testing. Additionally, we examined other morphological qEEG characteristics of SW, sleep spindles, and the phase-amplitude coupling between SW and spindles.

## Methods

### Participants

**Figure 1:**
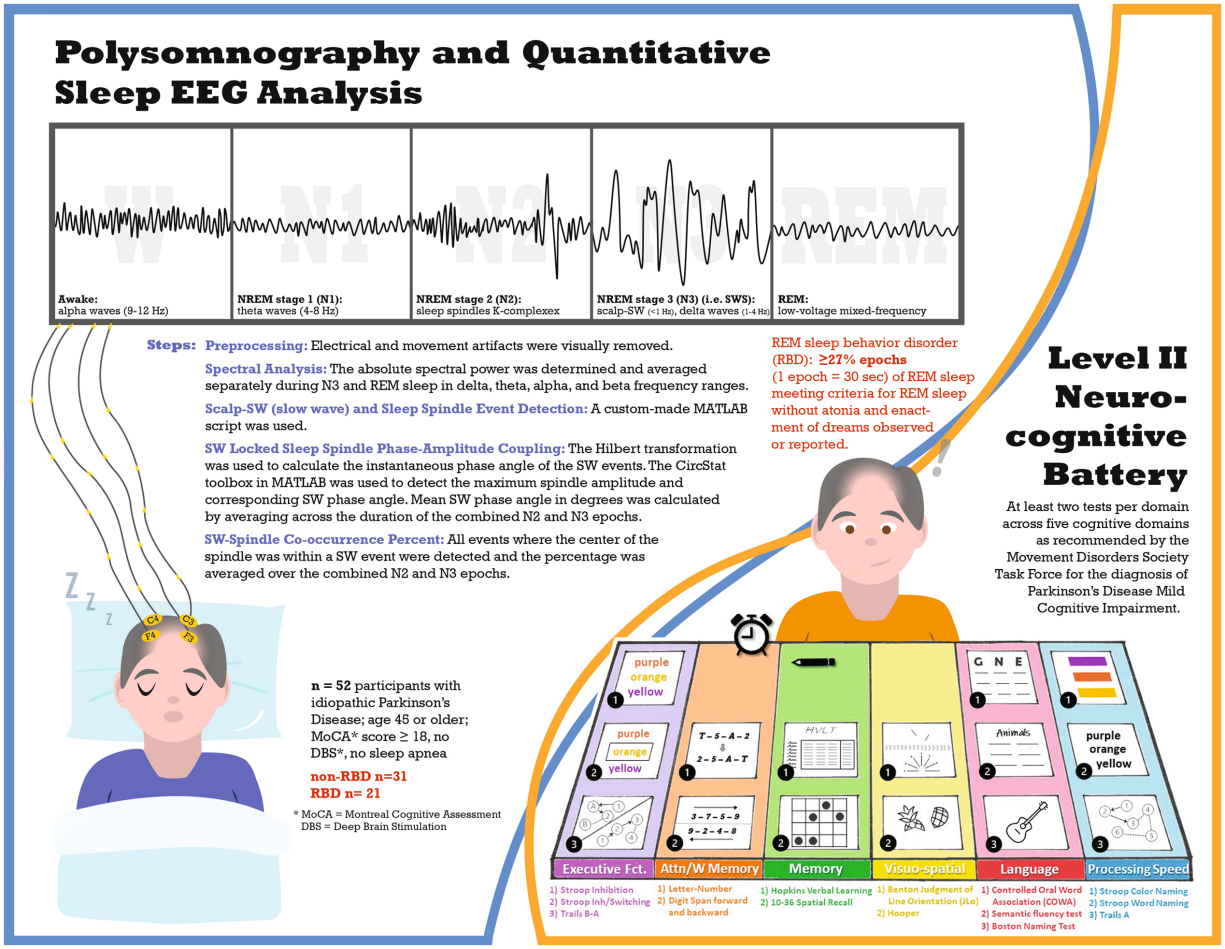
Infographic schematic of the study.

This cross-sectional study (see Figure 1 for study infographic) recruited participants from the University of Alabama at Birmingham (UAB) movement disorders clinic, who were part of a study on sleep and cognitive dysfunction in PD (4). All participants completed a level II neurocognitive assessment, as recommended by the Movement Disorders Society Task Force for diagnosis of PD-mild cognitive impairment (MCI) (19), and one night of polysomnography at UAB Sleep/Wake Disorders Center. Participants were eligible if they met the following criteria: (1) idiopathic Parkinson’s disease diagnosis as established by the Movement Disorders Society (20), (2) age 45 or older, (3) stable medication for at least four weeks before enrollment, and (4) Montreal Cognitive Assessment (MoCA) score of at least 18. Exclusion criteria included: (1) untreated or undertreated sleep apnea (apnea hypopnea index: AHI ≥5 events per hour), (2) atypical Parkinsonism, or (3) deep brain stimulation. UAB Institutional Review Board approved this study, and all participants provided written informed consent.

### Polysomnography

Supervised, laboratory-based polysomnography (PSG) was performed to obtain objective measures of sleep architecture including electroencephalograms (EEGs). The polysomnography procedure has previously been described (4). Each 30-s epoch of the recording was classified as wake, NREM stage 1 (N1), NREM stage 2 (N2), NREM stage 3 (N3) (i.e., SWS), or REM per the American Academy of Sleep Medicine scoring manual (21), REM sleep behavior disorder was defined as ≥27% epochs of REM sleep meeting criteria for REM sleep without atonia (22) plus enactment of dreams observed or reported (21).

### Quantitative sleep EEG analysis

#### Preprocessing

The PSG-derived EEG data were inspected for artifacts in 30-s epochs using MATLAB (version R2020b). The EEG evaluator (AAM) was blinded to the participant’s RBD status and performance on neurocognitive assessments. For the entire PSG recording, the F3 and C3 channels were visually evaluated, and electrical and movement artifacts were detected. In cases of continuous artifacts on F3/C3 leads, F4/C4 channels were used. The total artifact rejection included 2.3% of N2, 0.6% of N3, and 7.3% of REM for the PD-no-RBD group and 4.5% of N2, 1.1% of N3, and 13.4% of REM for the PD-RBD group. Because slow wave activity is most prominent over frontal regions during N3 and sleep spindle activity is most easily detected in central channels during N2 (14), SW and delta spectral power were averaged in the frontal lead during N3, while sleep spindles were averaged in the central lead during N2. In addition, SW-spindle coupling was computed across N2 and N3 in central EEG channels because the exact timing relationship is most prominent in the centro-parietal regions (23). Furthermore, spectral power during REM was measured in central leads to further minimize the effect of rapid eye movement artifact in frontal leads on power in the low frequency bands.

#### Spectral analysis

A Hamming window of 512 ms with 50% overlap was used to determine the spectral power with a resolution of 1 Hz. The absolute spectral power was determined and averaged separately during N3 and REM sleep in delta (1–4 Hz), theta (5–8 Hz), alpha (9–12 Hz), and beta (13–30 Hz) frequency ranges.

#### Scalp-SW and sleep spindle event detection

A custom-made MATLAB script was used to detect separate events for SW and spindles by applying well-established methods (23, 24). To identify SW, F3 (or F4 if F3 had continuous artifacts) was used and all zero crossings were identified. The following parameters were used for capturing SW events: (1) frequency filter = 0.16–1.25 Hz, (2) duration = 0.8–2 s, and (3) amplitude threshold = 75th percentile. We subsequently computed and averaged three morphological characteristics of SW across all N3 epochs: (1) density (events/min), (2) amplitude (peak to peak, expressed in μV), and (3) slope (expressed in μV per millisecond).

The following parameters were used to detect sleep spindles: (1) frequency filter = 9–15 Hz, (2) amplitude threshold = 75th percentile, (3) duration = 0.5–3 s. In those events that met the above criteria, the analytical amplitude was calculated using Hilbert’s transformation. Lastly, the following sleep spindle morphological characteristics were computed and averaged over all N2 epochs: (1) density (events/min), and (2) amplitude (peak to peak, expressed in μV).

#### SW locked sleep spindle phase-amplitude coupling

By applying the parameters discussed above, we first detected SW events. The Hilbert transformation was used to calculate the instantaneous phase angle of the SW events. We then filtered the same events between 9–15 Hz (spindle frequency) and derived the instantaneous amplitude by applying the Hilbert transformation. Using the CircStat toolbox in MATLAB (25), the maximum spindle amplitude and corresponding SW phase angle were detected (24). Mean SW phase angle in degrees was calculated by averaging across the duration of the combined N2 and N3 epochs.

#### SW-spindle co-occurrence percent

We calculated the co-occurrence percentage of the SW-spindle by detecting all events where the center of the spindle was within a SW event and then averaging the percentage over the combined N2 and N3 epochs.

### Level II neurocognitive battery

The neurocognitive battery included at least two tests per domain across five cognitive domains as recommended by the Movement Disorders Society Task Force for the diagnosis of PD-MCI (19). Neurocognitive battery assessments have been described in detail previously (4). The following tests were included in each domain: *Executive function*: (a) Delis-Kaplan Executive Function System (D-KEFS) Stroop color-word interference test: Stroop Inhibition, (b) D-KEFS Stroop inhibition/switching, and (c) Trails B-A; *Attention/Working Memory*: (a) Letter number sequencing of the Wechsler Adult Intelligence Scale-IV (WAIS-IV), and (b) Digit span forward and (c) backward of the Wechsler Memory Scale-III; *Memory*: (a) Hopkins Verbal Learning (HVLT) total recall and delayed recall; and (b) 10–36 Spatial Recall Test immediate and delayed; *Language*: (a) Controlled oral word association (COWA); (b) semantic/category fluency test, animals; and (c) Boston Naming Test (BNT); *Visuospatial Function*: (a) Benton Judgment of Line Orientation; and (b) Hooper Visual Organization Test. An additional domain, *Processing speed*, included (a) Stroop color-naming; (b) Stroop word naming; and (c) Trails A. Each cognitive test was converted to a normalized score (*z*-score) based on normative population values that were adjusted for age, sex, race, and educational level as appropriate. *Z*-scores for each test within a domain were averaged to determine the domain score, and domain scores were averaged to determine the comprehensive cognitive score (CCS).

### Statistical analysis

Statistical analysis was performed with JMP Statistical Discovery Pro version 16.0 and MATLAB version R2020b (for mean circular direction differences). We calculated and tested the descriptive statistics for normality using the Shapiro–Wilk test. For normally distributed data, we calculated the mean and standard deviation; otherwise, we reported the median and interquartile range. We compared the demographic, polysomnographic, and qEEG characteristics between the PD-no-RBD group and the PD-RBD group using Fischer’s test for categorical variables and Welch’s unequal variance two-tailed *t*-test for continuous variables, as appropriate. To confirm the validity of these results, we also performed logistic regression with RBD as the dependent variable and qEEG outcomes as predictor variables. We also performed stepwise multiple linear regression analysis to evaluate predictors of cognitive performance as measured by the CCS. In these models, CCS was the dependent variable, and we applied a forward selection procedure that included age, sex, education, MDS-UPDRS total score, levodopa equivalent dose, RBD, spindle density, SW density, and SW-spindle co-occurrence percent as potential predictors of CCS. We then performed a sensitivity analysis using a backward selection to verify the consistency of selection of the predictors. To account for multiple comparisons, value of *p*s were adjusted according to the Benjamini-Hochberg method (26). Adjusted value of *p*s (p’) <0.05 were considered significant.

## Results

### Participant characteristics

Table 1 summarizes the demographic, polysomnographic, and disease characteristics of participants. There were no statistically significant differences in any of the variables.

**Table 1:** Demographics, clinical, and polysomnographic characteristics.

Characteristics	PD-non-RBD	PD-RBD	F or Z ratio/U	*p*-value
*N*	31	21		
Age (years)				
Mean ± SD	64.9 ± 8.2	68.1 ± 5.4	2.9	0.09
Range	45–84	59–78		
Sex: *N* (%)				
Male	21 (67.7)	12 (57.1)	0.6	0.43
Female	10 (32.3)	9 (42.9)		
Race: *N* (%)				
Caucasian	29 (93.5)	20 (95.2)	0.07	0.8
African American	2 (6.5)	1 (4.8)		
Education (years)				
Median (IQR)	16 (14.0–18.0)	16 (14.5–17.5)	0.82	0.41
Medications that affect sleep: *N* (%)	20 (64.5)	13 (65.0)	0.001	0.97
Benzodiazepines	8 (25.8)	4 (19.1)	0.34	0.57
Duration of Disease (DOD) (years)				
Median (IQR)	4 (1.0–8.0)	6 (2.0–10.0)	1.1	0.28
Hoehn & Yahr *N* (%)				
1	4 (12.9)	1 (4.8)	1.8	0.41
2	23 (74.2)	15 (71.4)		
3	4 (12.9)	5 (23.8)		
Levodopa Equivalent Dose (LED)				
Median (IQR)	500.0 (260.0–765.0)	641.3 (395.0–1207.0)	1.6	0.11
MDS-UPDRS (Total)				
Mean + SD	51.0 ± 17.3	61.0 ± 20.7	3.4	0.075
Range	17–83	17–105		
Sleep Efficiency (%)	78.5 (71.6–86.1)	80.9 (67.3–87.7)	0.24	0.81
Total Sleep Time (min)	375.3 ± 52.6	373.2 ± 62.6	0.02	0.9
Wake After Sleep Onset (WASO) (min)	81.0 (61.5–115.1)	79.0 (52.7–143.9)	−0.18	0.86
Sleep Latency (min)	6.7 (3.9–18.5)	9.3 (5.6–12.8)	0.44	0.65
N1%	10.2 (8.1–14.2)	8.1 (5.9–16.9)	−0.94	0.35
N1 time (min)	38.5 (29.5–51.0)	31.5 (21.8–62.0)	−0.89	0.37
N2%	58.1 ± 8.3	55.7 ± 12.6	0.57	0.45
N2 time (min)	218.4 ± 47.6	209.0 ± 61.8	0.35	0.55
N3%	11.5 (6.4–23.0)	13.6 (5.1–26.6)	0.11	0.91
N3 time (min)	46.0 (23.0–86.5)	50.0 (19.0–106.5)	0.22	0.82
REM %	15.9 ± 5.8	14.8 ± 7.8	0.34	0.56
REM time (min)	60.1 ± 22.5	54.4 ± 29.5	0.57	0.45
Arousal Index	4.1 (3.4–6.3)	3.3 (2.1–5.9)	0.14	0.72
Apnea Hypopnea Index (events per hour)	0.3 (0.0–1.7)	0.4 (0.0–1.2)	−0.47	0.64
Periodic Limb Movements of Sleep	1.1 (0.1–14.1)	2.7 (0.3–24.0)	0.84	0.4

Mean ± SD presented for normally distributed data. Median (IQR) reported for non-normally distributed data. N1, non-REM stage1; N2, Non-REM stage 2; N3, non-REM stage 3; REM, rapid eye movement sleep. Medications potentially affecting sleep include benzodiazepines, non-benzodiazepine hypnotics narcotics, melatonin, trazodone, and gabapentin.

### Quantitative sleep EEG analysis

#### No differences in slow-wave morphology or delta spectral power during N3

**Figure 2:**
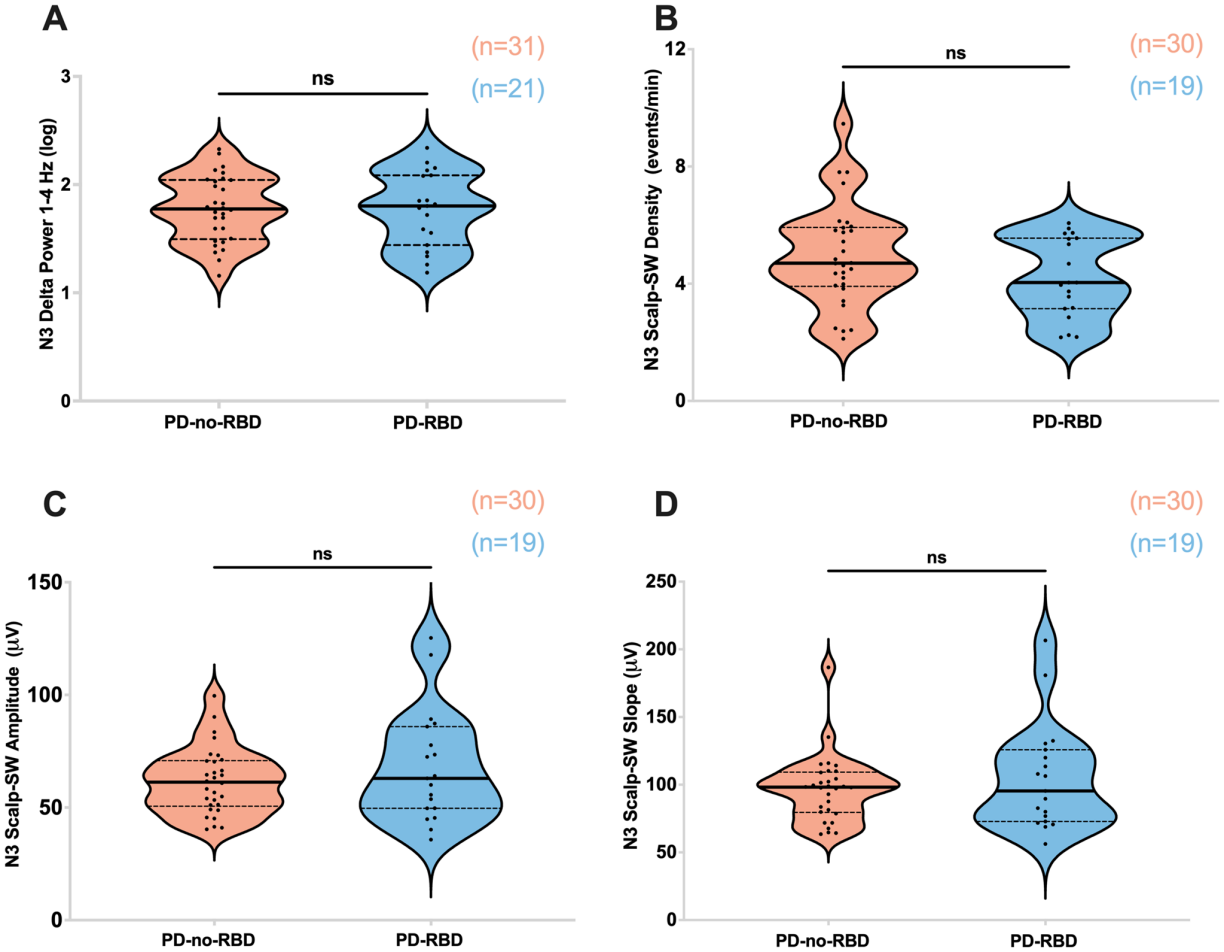
Absolute delta spectral power during N3 **(A)**, SW density **(B)**, SW amplitude **(C)**, and SW slope **(D)** were not different between PD-no-RBD and PD-RBD groups. The statistical analysis employed Welch’s unequal variance *t*-test. The graph displays dark bold lines indicating the median, while dotted lines represent the first and third quartiles. Individual values are denoted by dots. Data are displayed as violin plots. ns, not significant.

First, our objective was to assess whether the presence of RBD would influence slow wave morphology and delta spectral power, as these sleep parameters have significant implications for aging and cognitive impairment (27, 28). There were no significant differences in delta spectral power, SW density, peak-to-peak amplitude, or slope (amplitude divided by the time between SW peak and trough) between PD-RBD and PD-no-RBD (Figures 2A–D). In addition, no significant differences in log power spectra up to 30 Hz during N3 was found as shown in Supplementary Figure S1.

### Sleep spindles were reduced in PD-RBD group during N2 sleep

**Figure 3:**
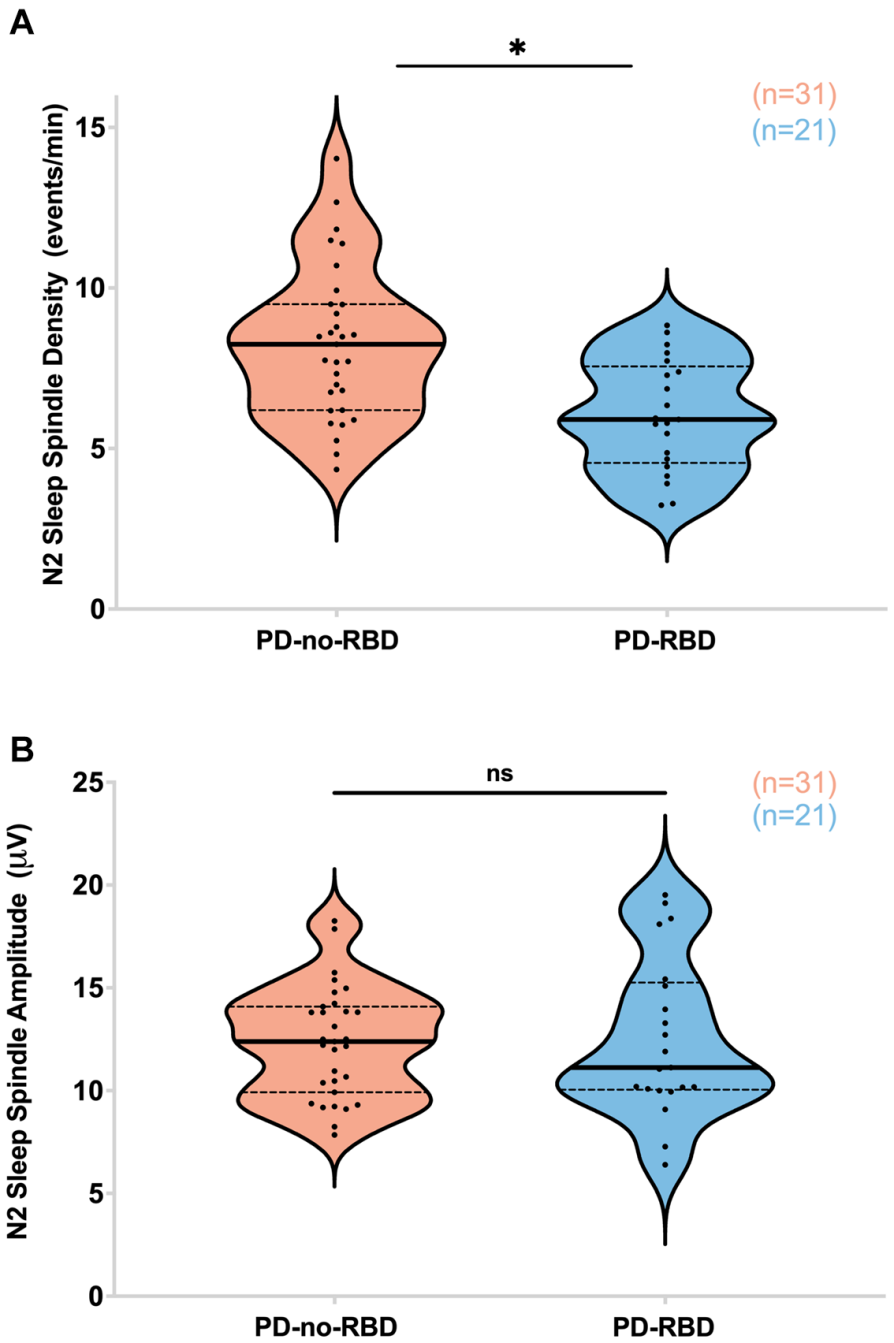
PD-RBD participants have lower spindle density during N2 compared to PD-no-RBD **(A)**. The spindle amplitude was not different between the two groups **(B)**. The statistical analysis employed Welch’s unequal variance *t*-test. The graph displays dark bold lines indicating the median, while dotted lines represent the first and third quartiles. Individual values are denoted by dots. Data are displayed as violin plots. ns, not significant; *p’: 0.0039.

Next, our objective was to examine the impact of RBD on sleep spindles, as sleep spindles are crucial for memory consolidation, and previous studies have indicated that they can predict the later development of dementia in Parkinson’s disease (10, 29–31). PD participants with RBD had significantly lower sleep spindle density (spindles/min of N2) compared to the PD-no-RBD group (*F* = 15.5, *p*’ = 0.0039) (Figure 3A). To confirm these relationships, logistic regression with RBD as the dependent variable and spindle density as the predictor variable showed that spindle density was a significant predictor of RBD (*χ*^2^ = 13.3, *p* = 0.0003). Spindle density remained a significant predictor of RBD when age was included in the model. To make sure benzodiazepines were not driving the differences between the groups, we repeated the analysis with exclusion of the 8 PD-no-RBD participants and the 4 PD-RBD participants who were taking benzodiazepines. With exclusion of those participants, the PD-RBD group (*n* = 17) still had significantly lower sleep spindle density (*F* = 19.9, *p* < 0.0001) compared to PD-no-RBD (*n* = 23). There were no significant differences between the two groups in spindle amplitude (Figure 3B). Upon further exploratory analysis, we observed that this finding remained consistent when we combined N2 and N3 sleep stages and when we divided spindles into slow (9–12 Hz) and fast (12–15 Hz) spindles, with lower spindle density in the PD-RBD group in all analyses (data not shown).

### SW-spindle co-occurrence percent was lower in PD-RBD group during combined N2 and N3 sleep

**Figure 4:**
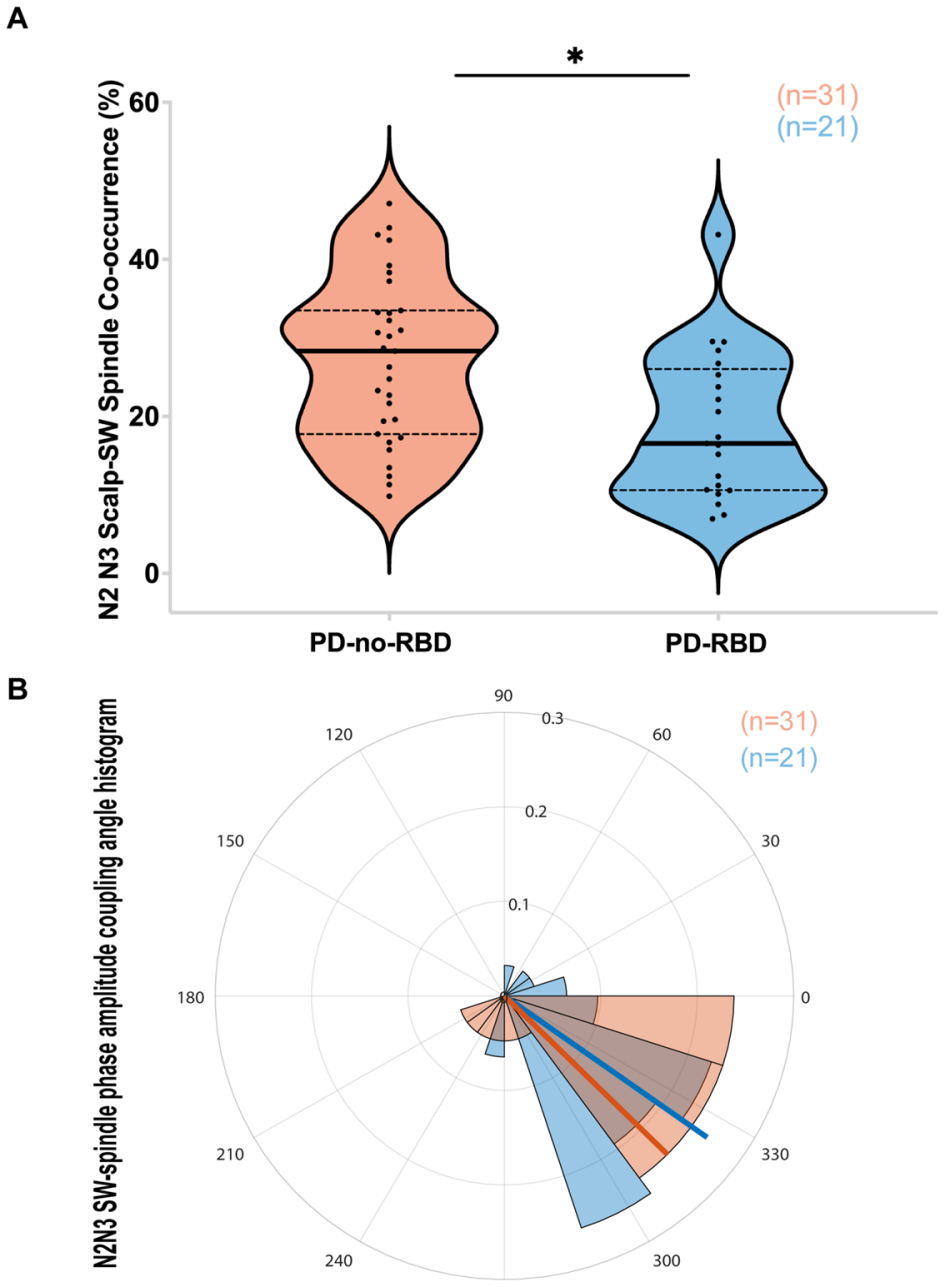
PD-RBD participants have lower SW-spindle co-occurrence percent compared to PD-no-RBD **(A)**. The statistical analysis employed Welch’s unequal variance *t*-test. The graph displays dark bold lines indicating the median, while dotted lines represent the first and third quartiles. Individual values are denoted by dots. The SW-spindle coupling mean angle **(B)** was not different between the groups using the CircStat toolbox to calculate mean circular direction differences. An angle value of 0 indicates synchrony, whereas 180 degrees indicates an anti-phase relationship. A value slightly below 0 (close to 360 degrees) suggests that spindle amplitude tended to peak shortly before the SW peak. The radius on the plot represents the strength of locking (coupling) between SW and spindles, shown in radians (0.1, 0.2, and 0.3). ns, not significant; *p’: 0.015.

We next examined whether SW-spindle phase-amplitude coupling would be reduced in PD-RBD, considering its potential significance for neural plasticity and memory consolidation (23, 32). The SW-spindle co-occurrence percent was significantly lower in the PD-RBD group than in the PD-no-RBD group (*F* = 9.44, p’ = 0.0156) (Figure 4A). Logistic regression with RBD as the dependent variable and SW-spindle co-occurrence percent as the predictor variable showed that SW-spindle phase amplitude coupling was a significant predictor of RBD (*χ*^2^ = 8.66, *p* = 0.0033). SW-spindle co-occurrence percent remained a significant predictor of RBD when age was included in the model. The mean coupling angle between the two groups did not differ significantly (Figure 4B). When excluding participants who were using benzodiazepines, the group with PD-RBD still had lower SW-spindle co-occurrence percent compared to the PD-no-RBD group (*F* = 37.9, *p* = 0.0003).

#### High theta spectral power during REM

**Figure 5:**
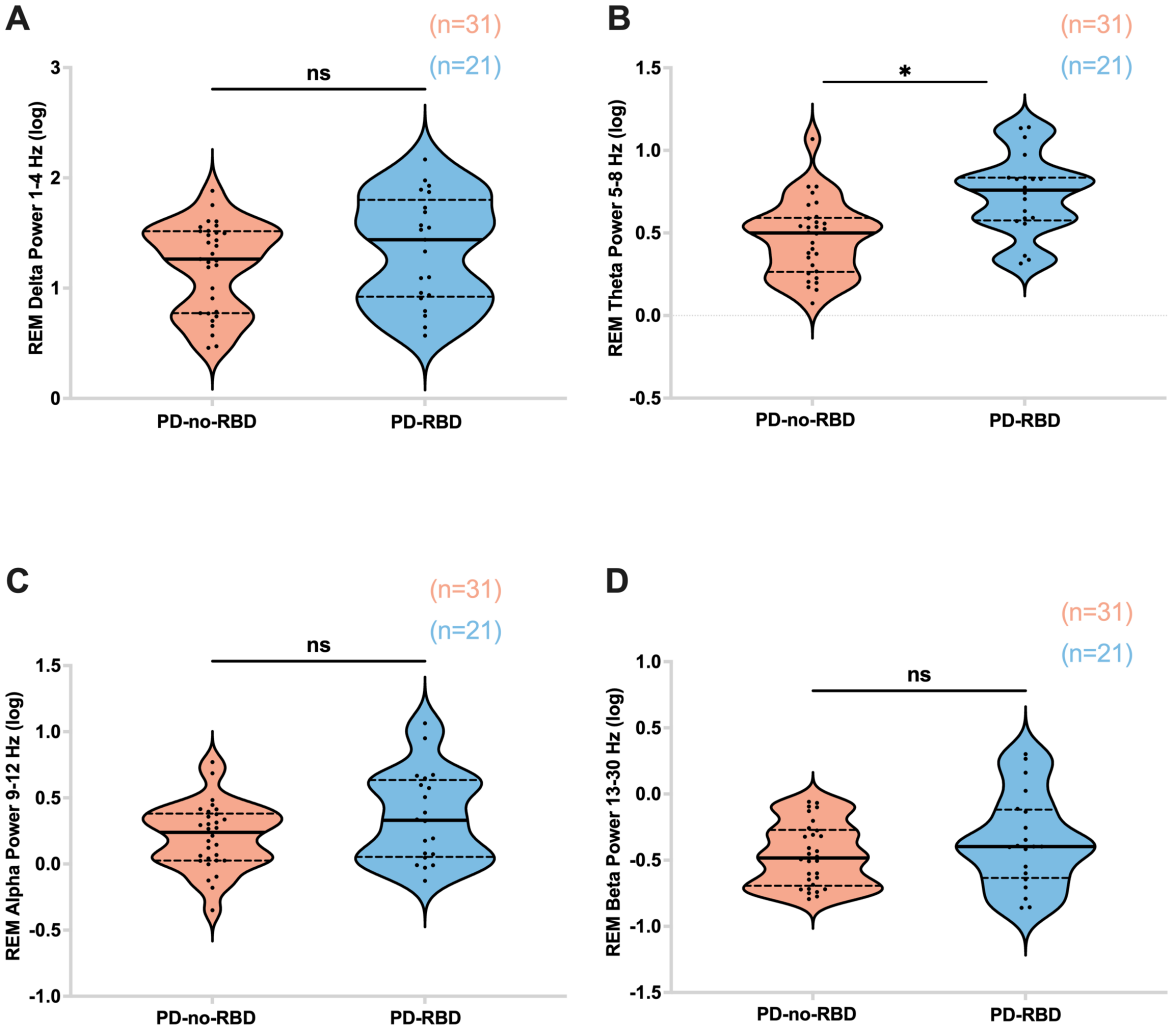
Absolute theta spectral power during REM is higher in the PD-RBD group compared to the PD-no-RBD group **(B)**. The spectral power in delta **(A)**, alpha **(C)**, and beta **(D)** were not different between the groups. Data are displayed as violin plots. The statistical analysis employed Welch’s unequal variance *t*-test. The graph displays dark bold lines indicating the median, while dotted lines represent the first and third quartiles. Individual values are denoted by dots. ns, not significant; *p’: 0.0039.

REM theta power was significantly higher in the PD-RBD group compared to the PD-no-RBD group (*F* = 3.59, *p*’ = 0.0039). Logistic regression with RBD as the dependent variable and REM theta spectral power as the predictor variable showed that theta power during REM was a significant predictor of RBD (*χ*^2^ = 12.17, *p* = 0.0005). This remained significant when age was included in the model. However, there were no significant differences between the PD-no-RBD and PD-RBD groups for power in the delta, alpha, or beta spectral frequency (all p’ > 0.05) (Figure 5). Supplementary Figure S2 shows the central EEG log power spectrum during REM up to 35 Hz.

### Impaired cognitive performance in PD-RBD group

**Figure 6:**
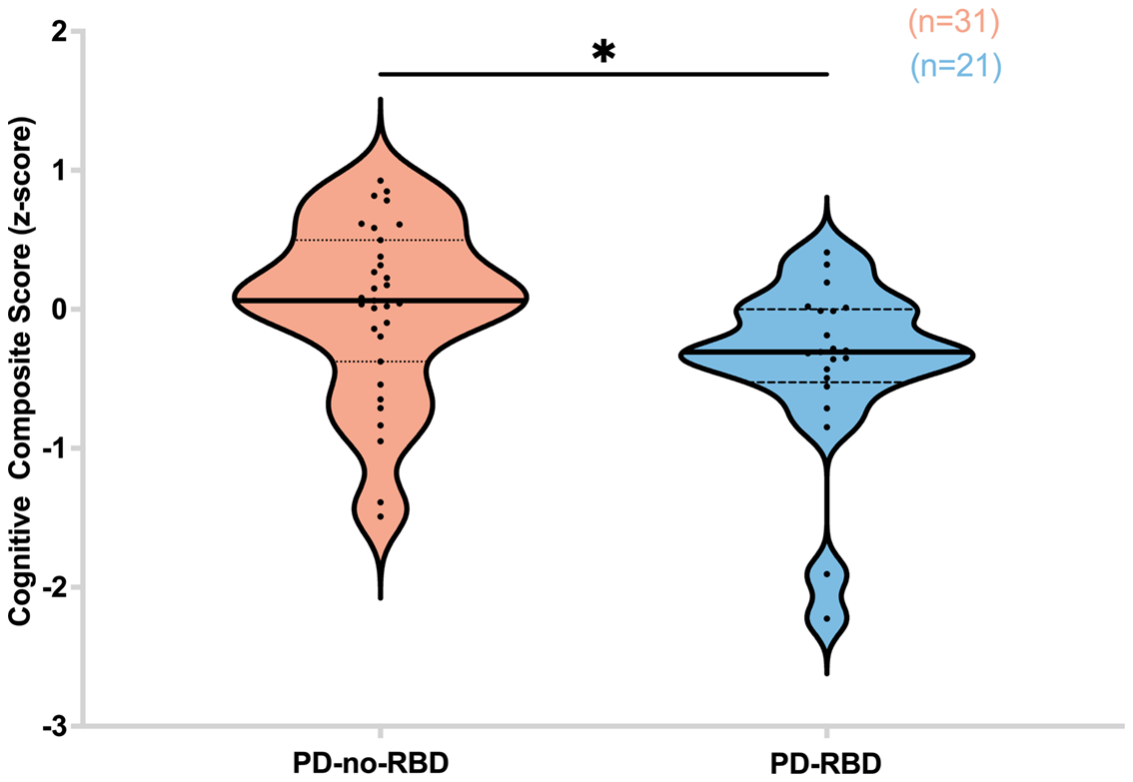
Cognitive composite score (*z*-score) is lower in the PD-RBD group compared to the PD-non-RBD group. Data are displayed as violin plots. The statistical analysis employed Welch’s unequal variance t-test. The graph displays dark bold lines indicating the median, while dotted lines represent the first and third quartiles. Individual values are denoted by dots. *p’: 0.047.

Subsequently, we investigated the potential influence of RBD on cognitive performance in Parkinson’s disease, considering the association of iRBD with cognitive decline. Our findings revealed that in the PD-RBD group, the CCS score was significantly lower than in the PD-no-RBD group (*F* = −2.44, p’ = 0.047) (Figure 6).

#### Predictors of cognitive performance

In a stepwise multiple regression model using a forward selection procedure, two variables were significant predictors of cognitive performance as measured by the CCS. The strongest predictor was sleep spindle density, accounting for 17% of the variance (*β* = 0.12, *t* = 4.05, *p* = 0.0002) followed by education, which accounted for an additional 8% of the variance (*β* = 0.08, *t* = 2.56, *p* = 0.0134). Presence of RBD and percentage of REM sleep without atonia were not significant predictors of CCS. As a sensitivity analysis, we ran the same model using all variables with a backward selection procedure, which confirmed that these predictors were independent of method of selection.

## Discussion

This study examined the differences in sleep-related qEEG oscillations between individuals with PD-RBD versus PD-no-RBD and investigated the relationship between sleep qEEG characteristics and cognitive performance. These findings add to the growing evidence supporting sleep neurophysiology’s role in brain plasticity and cognition (14, 16, 24). Specifically, we found that individuals with PD and RBD have lower sleep spindle density, lower SW-spindle phase amplitude coupling percent, higher REM theta spectral power, and lower CCS scores than PD participants without RBD. Intriguingly, although RBD is a REM parasomnia (14), the present study found that sleep qEEG signatures of NREM sleep are also altered in PD-RBD patients, possibly related to the worse cognitive performance. These results extend previous work showing lower spindle density and poorer cognitive performance in iRBD patients compared with non-RBD controls (3). Further, our findings suggest that RBD-associated reduction in sleep spindle density may contribute to the lower cognitive performance among PD patients with RBD compared to those who do not have RBD.

Sleep spindles are produced in the thalamus and synchronized in the cortex, demonstrating the efficiency of the thalamocortical system (14). This study found that PD-RBD patients had lower spindle density compared to PD-no-RBD, but there were no group level differences in spindle amplitude. These results are similar to previous findings of decreased spindle density (3) in iRBD compared to controls, suggesting possible deficits in corticothalamic circuits in RBD. Indeed, a retrospective study from the Parkinson’s Progression Markers Initiative (PPMI) evaluating structural brain characteristics in *de novo* PD patients with probable RBD (pRBD) found lower thalamic volume in the PD-pRBD group compared to PD-no-pRBD group (7). Further research could investigate the relationship between thalamic volumetric analysis and spindle density.

According to the “active system consolidation” theory, sleep spindles play a crucial role in declarative and non-declarative forms of memory through synaptic plasticity (14). According to prior longitudinal studies, iRBD is an important clinical risk factor for cognitive decline in individuals with PD (8, 33). In our study, using regression analysis to predict global cognitive performance revealed that spindle density is a significant predictor of cognitive performance, suggesting spindle density as an underlying mechanism to explain the faster cognitive decline in PD-RBD patients compared to PD-no-RBD patients (7). This compelling hypothesis needs to be further explored in longitudinal studies.

In this comparison of PD-RBD to PD-no-RBD, there were no significant differences in SW density or morphology (amplitude and slope), or in delta spectral power during N3, suggesting that in this PD cohort RBD status did not further impair the slow wave activity dynamics at the neuronal network or structural levels as these markers are altered in aging and cognitive impairment (28). This is similar to the findings by Latreille and colleagues, who found no significant differences in delta power and SW characteristics between iRBD and control groups during NREM sleep (34). Interestingly, Sunwoo and colleagues found that patients with iRBD had a lower SW slope compared to controls (2). Based on a previous study evaluating the association between SW slopes and white matter diffusion in healthy adults, the authors speculated that a steeper slope of SWs might indicate a decline in axonal integrity and decreased connectivity in the frontal cortex (35). This, in combination with findings of lower delta power in newly diagnosed PD patients compared to controls (36), suggests that the PD disease pathology rather than presence of RBD pathology influences delta power in N3. Additional study of SW morphology in PD compared to controls is needed to fully understand these relationships.

A recent case–control study investigated the impact of iRBD pathology on SW-spindle coupling, finding that patients with iRBD had misaligned SW-spindle coupling (2). The pattern of misaligned coupling observed during the transition from the down-to-up state of SW is consistent with that seen in older adults relative to younger adults (23), suggesting that iRBD patients are experiencing an accelerated process of aging or degeneration. The current study indicates for the first time that PD patients with RBD pathology also have a lower percentage of SW-spindle co-occurrence. However, no significant differences were detected in terms of SW-spindle mean coupling angle. Given that spindles are generated by the cortico-thalamocortical pathway (37), the reduced coupling percent in the PD-RBD group suggests possible neurodegeneration in cortico-thalamocortical networks.

During REM sleep, we found higher theta spectral power in PD-RBD compared to PD-no-RBD. Although cholinergic activity in NREM sleep is reduced, cholinergic signaling increases during and regulates REM sleep (38). Studies have also demonstrated reduced volumes of cholinergic basal forebrain nuclei in PD patients with cognitive impairment (39–41), possibly leading to an upregulation of cholinergic receptors in response to cholinergic neuron loss (42–44). Additionally, the role of GABAergic neurons in the basal forebrain deserves mention, as they play a significant role in influencing theta oscillations (45) and spindle activity (46, 47). These neurons have been identified as essential regulators and generators of both theta oscillations and regular sleep spindles, contributing to the modulation of the sleep–wake cycle (46). In one case–control study of 61 iRBD patients and 28 control subjects, the absolute delta and theta power were elevated in the iRBD group, indicating early neurodegeneration impacting cortical and subcortical cholinergic transmission in RBD patients (17). To the best of our knowledge, the present study is the first to examine REM spectral power in PD-no-RBD and PD-RBD groups. The finding that PD-RBD patients have a higher absolute power in the theta frequency range suggests that REM theta power alterations could also act as a potential marker of neurodegeneration in the PD-RBD group and should be further investigated in longitudinal studies with a control group.

This study has several strengths, including the comprehensive qEEG sleep analysis, the blinding of the EEG analysis to group (PD-RBD vs. PD-no-RBD), correction for multiple comparisons, and the comprehensive cognitive assessment. However, there are limitations to this study. First, we did not account for the first-night effect (poor sleep in an unfamiliar setting). However, in our prior research, the first-night effect was not observed to affect sleep in patients with PD and if this were a factor, would be expected to affect both groups equally. Second, we did not have a control group that was not affected by PD or RBD, which would have allowed investigation of PD-specific effects on sleep qEEG. This can be explored in future studies. Finally, we did not exclude participants who were on medications that affect sleep. However, there was no significant difference between the groups in the number of individuals taking such medications (*p* = 0.97).

In conclusion, this study demonstrates a lower sleep spindle density, lower SW-spindle co-occurrence percent, higher theta spectral power during REM, and poorer cognitive performance in PD-RBD patients compared to PD-no-RBD patients. Further, the findings suggest that reduction in sleep spindle density may be one mechanism through which RBD is associated with worse cognition in patients with PD. This study is novel because the influence of RBD in PD is examined quantitatively in all sleep stages. These findings provide a rationale for conducting future longitudinal studies to determine whether sleep spindle density may contribute to the development of more rapid cognitive decline in patients with PD-RBD.

## Data availability statement

The original contributions presented in the study are included in the article/Supplementary material, further inquiries can be directed to the corresponding author.

## Ethics statement

The studies involving humans were approved by the University of Alabama at Birmingham. The studies were conducted in accordance with the local legislation and institutional requirements. The participants provided their written informed consent to participate in this study.

## Author contributions

AM, ZI, SM, and AA were involved in conceptualization of the research project. AM, CC, JP, RM, AJ, KW, and AA were involved in organization of the research project. AM, CC, ZI, RM, AJ, KW, and AA were involved in execution of the research project. AM, KW, GC, and AA were involved in execution of the statistical analysis. AM, CC, ZI, RM, AJ, KW, GC, SM, and AA were involved in review and critique of statistical analysis. AM wrote the first draft. AM, CC, ZI, JP, RM, AJ, KW, GC, SM, and AA were involved in review and critique of the manuscript. All authors contributed to the article and approved the submitted version.

## Funding

AM received funding from NINDS R25: NS079188 Training Fellowship; KW received funding from NIH: T32 HD071866 Training Fellowship; AA received funding from NIH (K23NS080912 and R01HD100670). SM received funding from NIH (K23NS097576). There are no additional disclosures by the authors that are relevant to this manuscript.

## Conflict of interest

The authors declare that the research was conducted in the absence of any commercial or financial relationships that could be construed as a potential conflict of interest.

## Publisher’s note

All claims expressed in this article are solely those of the authors and do not necessarily represent those of their affiliated organizations, or those of the publisher, the editors and the reviewers. Any product that may be evaluated in this article, or claim that may be made by its manufacturer, is not guaranteed or endorsed by the publisher.

## Supplementary material

SUPPLEMENTARY Figure 1 Frontal EEG log power spectrum during N3. No significant statistical difference was observed with Welch’s two-tailed *t*-test. *p*’: adjusted *p*-value.

SUPPLEMENTARY FIGURE 2 Central EEG log power spectrum during REM. REM theta power was significantly higher in the PD-RBD group using Welch’s two-tailed *t*-test. No statistical difference was found in other frequency bands *p*’: 0.0039.
