# Supplementary figures and images for: Quantitative sleep electroencephalogram and cognitive performance in Parkinson’s disease with and without rapid eye movement sleep behavior disorder
Source: Front Neurol. 2023 Sep 7;14:1223974. doi: 10.3389/fneur.2023.1223974 (PMC10512724; doi:10.3389/fneur.2023.1223974)

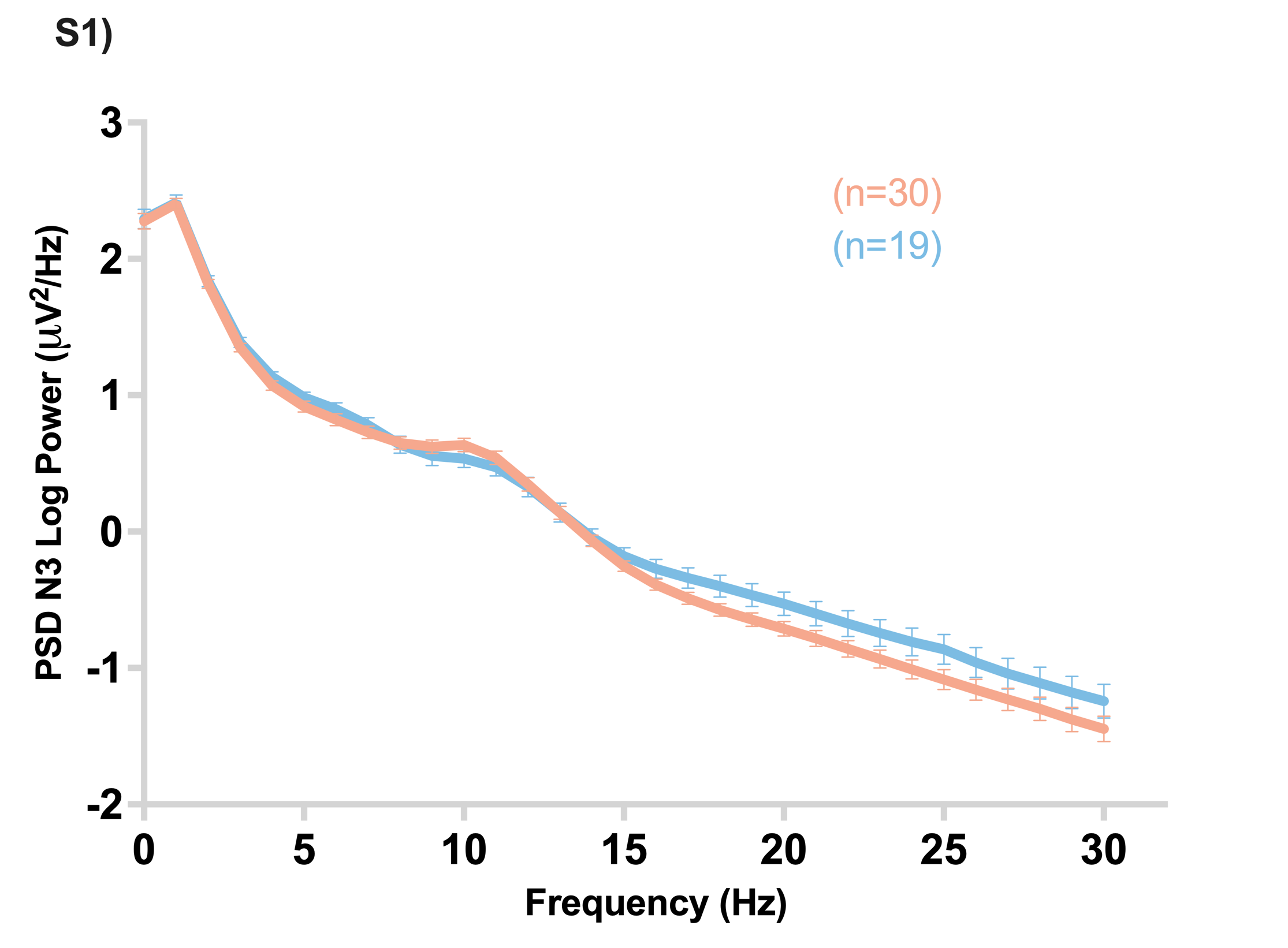

Supplement: SUPPLEMENTARY Figure 1 — Frontal EEG log power spectrum during N3. No significant statistical difference was observed with Welch’s two-tailed t-test. p’: adjusted p-value. [file Figure_S1.TIFF]

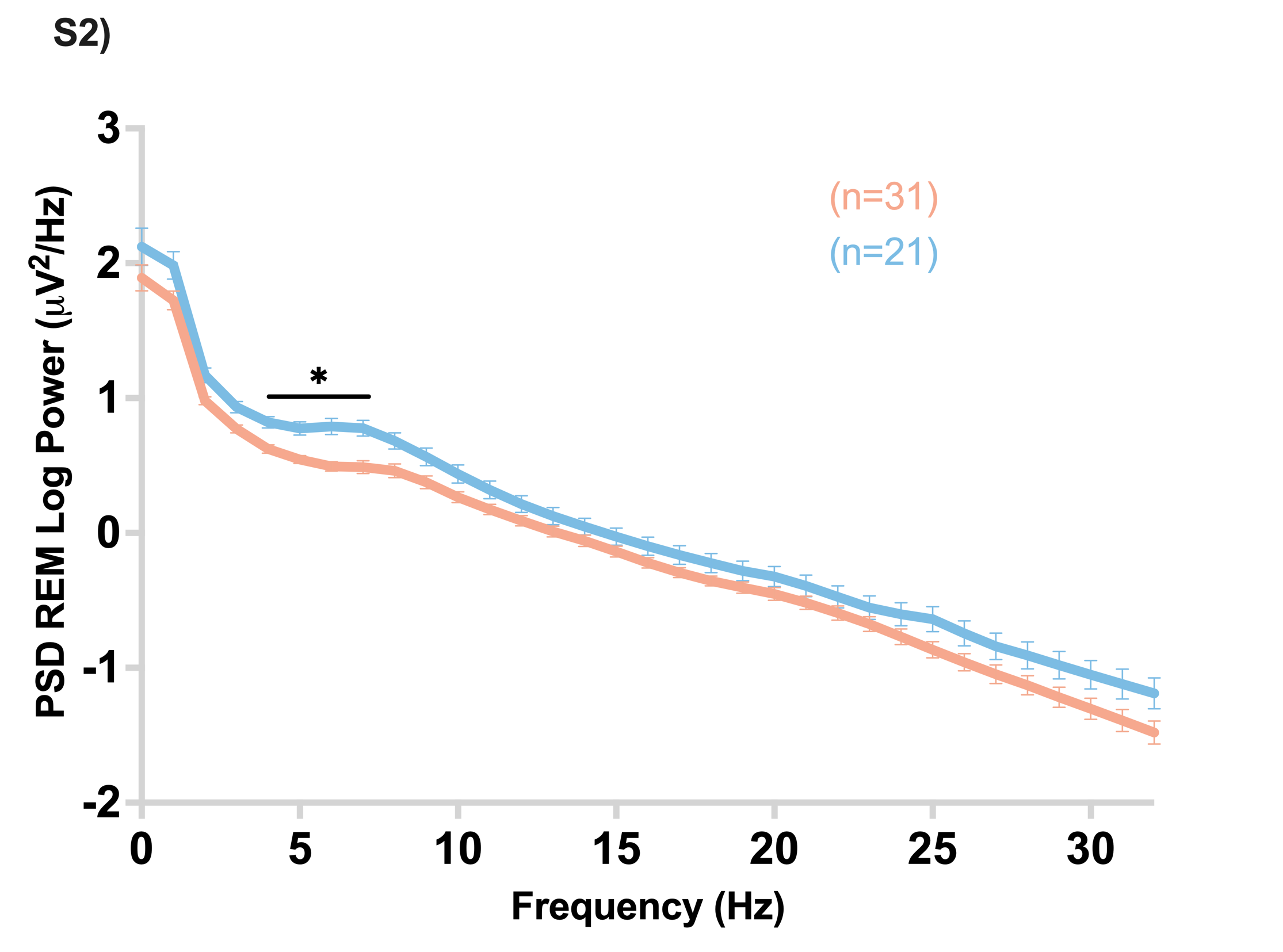

Supplement: SUPPLEMENTARY FIGURE 2 — Central EEG log power spectrum during REM. REM theta power was significantly higher in the PD-RBD group using Welch’s two-tailed t-test. No statistical difference was found in other frequency bands p’: 0.0039. [file Figure_S2.TIFF]
